# Validation of a Newly Developed Food Frequency Questionnaire to Assess Dietary Intakes of Magnesium

**DOI:** 10.3390/nu11112789

**Published:** 2019-11-15

**Authors:** Deeptha Sukumar, Rosemary DeLuccia, May Cheung, Rohit Ramadoss, Tammy Ng, Alicia Lamoureux

**Affiliations:** 1 Department of Nutrition Sciences, College of Nursing and Health Professions, Drexel University, 1601 Cherry St., Philadelphia, PA 19102, USA; rkd44@drexel.edu (R.D.); mmc372@drexel.edu (M.C.); rr627@drexel.edu (R.R.); a.m.lamoureux@gmail.com (A.L.); 2 College of Medicine, Drexel University, 2900 W Queen Lane, Philadelphia, PA 19129, USA; thn47@drexel.edu

**Keywords:** magnesium, food frequency questionnaire, food diary, validation study

## Abstract

Magnesium (Mg) intake is an important indication of an individual’s Mg status, but no validated food frequency questionnaire (FFQ) to assess intake currently exists. The purpose of this study was to develop and investigate the validity of a semi-quantitative Mg food frequency questionnaire (MgFFQ) against a 14-day food diary to assess average daily Mg intakes. In this cross-sectional study, 135 adults aged 18 to 75 completed the 33-item MgFFQ and a 14-day food diary to assess their Mg intakes. Coefficients of variance, Pearson’s correlation coefficients, and/or Spearman’s rank correlation coefficient tests were used to determine the relationship between the MgFFQ and the average Mg intake from the 14-day food diary among all participants, men, women, age groups, and body mass index (BMI) groups. The correlation between the MgFFQ and the 14-day food diary was significant (*p* < 0.05) for all participants (*r* = 0.798), men (*r* = 0.855), women (*r* = 0.759), normal weight (*r* = 0.762), overweight (*r* = 0.858), and obese (*r* = 0.675) weight statuses, and in all age groups. The calcium to magnesium intake (Ca:Mg) ratio in all participants was higher than optimal, 3.39 (2.11). Our results suggest that the MgFFQ is a valid method to capture Mg intake over an extended period of time, therefore acting as a valuable tool to quickly determine Mg intake.

## 1. Introduction

Magnesium (Mg), is the fourth most abundant mineral and second most abundant intracellular cation in the human body, acting as a cofactor for over 300 enzymatic reactions [1,2,3]. The biological functions of Mg in the body include but are not limited to improved adenosine triphosphate (ATP) production, deoxyribonucleic acid (DNA)/ribonucleic acid (RNA) synthesis, insulin sensitivity, bone health, and cardiovascular health [1,2,3,4,5]. Magnesium also plays an important role in vitamin D synthesis and metabolism in the body, thereby enhancing its function [6]. The maintenance of an adequate Mg status is integral to overall health, and the predominant way to improve Mg status is through an increase of dietary Mg intake [1]. Therefore, dietary Mg intake is an important consideration when evaluating an individual’s Mg status [1,2].

The Institute of Medicine Recommended Daily Allowance (RDA) for Mg for adults 31 to 70 years is 310 mg for women and 420 mg for men [2]. Magnesium is found in high amounts in food sources such as fruits, vegetables, and whole grains [2,4]. Conversely, processed foods tend to have a lower Mg content [2,4]. Under normal physiological circumstances, approximately 50% of dietary Mg can be absorbed into the human body; however, the biggest factor that determines the percent of Mg absorbed is an individual’s Mg status [7].

Individuals who are Mg deficient will have an increased intestinal absorption rate of Mg and a decreased urinary Mg excretion amount compared to individuals who are not deficient [7]. The Mg loading test, the gold standard for measuring Mg status, assesses an individual’s Mg status through measuring urinary Mg excretion after Mg infusion [8]. However, this infusion method is cumbersome and requires normal kidney and gastrointestinal functions for an accurate measure of Mg status [2,8,9]. Additionally, Mg status can be negatively affected by high calcium (Ca) status, leading to an antagonistic effect of this mineral on intestinal Mg absorption [10]. This is due to the a shared homeostatic regulating system involving Ca^2+^ sensing receptor (CaSR), and the affinity of Mg ion transporters (TRPM7) for Ca [10]. Consequently, a high serum Ca concentration reduces intestinal absorption rates for both Mg and Ca in humans, potentially exacerbating Mg insufficiency while causing further imbalance to the Ca:Mg intake ratio [10]. High dietary fiber, phosphorus, oxalic acid, and phytic acid consumption can also result in lower Mg absorption and status [11,12,13]. Furthermore, although commonly measured in a clinical setting, serum Mg concentrations are known to provide limited information on overall Mg status [2]. Due to these limitations in Mg status measurement, accurately capturing dietary Mg intake to assess Mg adequacy is an important tool to provide a comprehensive representation of an individual’s overall Mg status [7].

Food frequency questionnaires are convenient, low-cost tools that are widely used for assessing nutrient intakes in clinical research [14,15]. The current National Health and Nutrition Examination Survey (NHANES) Food Frequency Questionnaire (FFQ) is a semi-quantitative questionnaire that contains 139 questions designed to measure the intake of macro and micronutrients across the board [16]. Although this questionnaire is comprehensive, it is lengthy and not specific to Mg intake [16]. This may pose a heavy burden on individuals participating in the FFQ. Therefore, it may not be feasible and cost effective for clinical research studies. A validated, nutrient-specific FFQ may offer an efficient and cost-effective way to evaluate nutrient intakes for clinical research studies. While other FFQs have been developed and validated to assess different micronutrient intakes in various demographic populations such as calcium, vitamin D, and sodium, currently, no validated Mg-specific FFQ is available for this purpose [15,17,18].

The “gold standard” for measuring a dietary nutrient intake is through the use of multiple days of food diaries [19]. However, this method is labor intensive for both participants and researchers [19]. Furthermore, this method can only capture dietary intake over several days, rather than capturing typical overall daily Mg intakes that are more representative of long term dietary consumption habits [15,19]. The purpose of this study was to develop and determine the validity of a newly developed, semi-quantitative Mg food frequency questionnaire (MgFFQ) in comparison to a 14-day food diary for use in clinical research to easily assess average daily dietary Mg intakes.

## 2. Materials and Methods

### 2.1. Participant and Study Design

Participants were recruited within the Drexel University campus and in the surrounding community, and all individuals were screened for eligibility before participating in the study. Healthy individuals with no major diagnosed medical conditions or prescription use that may impact magnesium intake or absorption, between the ages of 18 to 75 years were eligible to participate in this study. Individuals who were unable to speak and understand English were excluded, since the MgFFQ being validated was in English. Prior to participation, all participants completed informed consent for the study in accordance with the Drexel University Institutional Review Board (IRB# 1501003336) for human research. This study was also registered on Clinicaltrials.gov (NCT03470519).

### 2.2. Magnesium Food Frequency Questionnaire

The MgFFQ is a 33-item, semi-quantitative tool specifically designed to measure Mg intake (Appendix A). The foods present on the MgFFQ were chosen from the United States Department of Agriculture’s National Nutrient Database, in accordance with commonly consumed foods in the United States of America that are considered good sources of Mg (>5% Daily Value), as well as foods that have lower amounts of Mg per serving, such as bread, which are often consumed in multiple servings, thus contributing a significant amount of Mg to the diet. Examples of foods on this questionnaire included dark leafy greens (kale, spinach, collards), nuts and seeds (cashews, pecans, pumpkin seeds, sunflower seeds), fishes (salmon, mackerel), grains (brown rice, whole wheat bread, white bread), and dairy products (yogurt, milk). Foods ranged from cashews, having the highest amount of Mg for one serving (178 mg), to white bread, having the lowest amount of Mg for one serving (6 mg).

### 2.3. Data Collection

#### 2.3.1. Orientation Visit

All eligible participants attended a 30-min orientation visit to begin study participation. During this visit, informed consent, demographic information, and the MgFFQ were completed for all participants. Additionally, in a subset of participants, an additional calcium food frequency questionnaire (CaFFQ) was completed. Demographics information that was self-reported from all participants included age, sex, race/ethnicity, height, weight, medical history, and current medications. Body mass index (BMI) was calculated from the height and weight information.

#### 2.3.2. Magnesium Food Frequency Questionnaire:

The MgFFQ was conducted by trained research personnel who verbally asked participants if they had consumed a certain food or beverage. If the answer was “yes”, the researcher determined the frequency and amount by asking “how often” and “how much” that certain food or beverage was consumed on average. Common serving sizes for each food or beverage were listed on the questionnaire to capture portion sizes, and Mg content for those serving sizes were also listed on the MgFFQ. The frequency of consumption the listed foods or beverage could be reported in were: “number of times per day”, “number of times per week”, or “number of times per month”. The response for quantity of consumption was open-ended for the participants. Upon questionnaire completion, the daily Mg intake per item was calculated by dividing the Mg content of each food or beverage item reported in a weekly or in a monthly consumption by 7 and 30, respectively. Then the average Mg intake per day, reported in milligrams (mg), was calculated by adding the “daily Mg intake per item” column on the questionnaire.

#### 2.3.3. Calcium Food Frequency Questionnaire

A previously validated CaFFQ was administered to each participant by trained research personnel in the same manner that the MgFFQ was administered [17]. This CaFFQ has been validated by the International Osteoporosis Foundation [17]. Upon CaFFQ completion, the daily Ca intake per item was calculated by dividing the Ca content of each food or beverage item reported in a weekly or in a monthly consumption by 7 and 30, respectively. Then the average Ca intake per day, reported in milligrams (mg), was calculated by adding the daily Ca intake per item column on the questionnaire. Daily Ca supplement use was also calculated using the CaFFQ.

#### 2.3.4. 14-Day Food Diary

A blank 14-day food diary was given to participants after the orientation visit, to be completed over the next consecutive 14 days [18]. During the baseline visit, all participants were instructed on how to use measuring cups and food scales to quantify their consumption, and an example of a day of food recording was completed. Participants were instructed to record all food and drinks consumed at all meals and snacks, with the exception of water. Information such as “food type”, “amount”, “methods of preparation”, “brand name of food and portion size”, and “who prepared meal” were asked. Participants were asked to record all foods and beverages consumed over a 24-h period for each food log day. After the 14-day recording period, participants returned their food diaries to the researchers, who reviewed them prior to analysis to assess accuracy and detail of recording. The food diaries were then analyzed using the FoodWorks version 17 software (Long Valley, NJ, USA) for nutritional analysis.

### 2.4. Statistical Analysis

Participants were classified by BMI groups accorded to standard categories [19], and by age groups of 18 to 29 years, 30 to 39 years, 40 to 49 years, 50 to 59 years, 60 to 69 years, and 70 to 75 years. Outliers >2.5 standard deviations from the mean were removed for all continuous variables. Normality was assessed for all variables with calories, protein, carbohydrates, sugar, cholesterol, phosphorus, potassium, thiamin, riboflavin, and vitamin B12 being normally distributed, and all other variables being not normally distributed. Means and standard deviations or medians and interquartile ranges were used to assess participant demographics, macronutrient, and micronutrient information. Coefficients of variance, Pearson’s correlation coefficient, and/or Spearman’s rank correlation coefficient tests were used to determine the relationship between the MgFFQ and the average Mg intake from the 14-day food diary among all participants, men, women, age groups, and BMI groups. Data were analyzed using IBM SPSS Statistics version 24.0 (IBM Corporation, Armonk, NY, USA). Level of significance was set to *p* < 0.05 prior to analysis.

## 3. Results

### 3.1. Demographics

A total of 135 participants participated in this study (Table 1) [18]. Of all the participants, there were significantly (*p* < 0.001) more women, 67.4%, than men, 32.6%, as deteremined by a Chi-square test for expected values. Sixty-three percent (63%) of the total sample were Caucasian, 25.2% were Asian, 5.2% were African American, 4.4% were Hispanic, and 2.2% were of other ethnicities. The average age for this study sample was 31.25 years, and was not significantly different between sexes, with an average age for women of 30.84 years, and an average age for men of 32.70 years. In all participants, 4.4% of the study sample had an underweight BMI, 52.6% had a normal weight BMI, 31.1% had an overweight BMI, and 11.9% had an obese BMI.

### 3.2. Dietary Intake

Dietary intake from the 14-day food diary was assessed for all participants (Table 2). Carbohydrates and protein intakes exceeded the RDA, but fell within the Acceptable Macronutrient Distribution Range (AMDR) for both women and men. Total fat intakes exceeded the RDA but fell within the AMDR for women, and exceeded the RDA and fell slightly higher than the AMDR for men. Saturated fat accounted for approximately 9.8% and 11.1% of total intake in women and men, respectively, and sugar accounted for approximately 14.1% and 12.8% of total intake in women and men, respectively. Fiber intakes fell considerably below the RDA in both women and men.

Micronutrient intake was largely insufficient in both sexes (Table 3). On average, both women and men consumed lower amounts of vitamin A, vitamin C, vitamin D, vitamin E, folate, and potassium than the RDAs for age and sex. Additionally, women consumed insufficient iron and men consumed insufficient zinc compared to the RDAs for sex and age. Vitamin K, vitamin B6, vitamin B12, thiamin, niacin, riboflavin, phosphorus, and sodium intakes were sufficient in both sexes. Additionally, intakes of zinc and iron were sufficient in women and men, respectively, compared to the RDAs for sex and age.

Both Ca and Mg intakes, measured by the 14-day food diary as well as the CaFFQ and MgFFQ, were insufficient in both sexes compared to the RDAs for age and sex (Table 4). Intake of Mg measured from the MgFFQ was approximately 40 mg less than the intake of Mg measured from the 14-day food diary in both sexes. Among women, 18.7% reported Ca supplement use, and among men 13.6% reported Ca supplement use.

The Ca to Mg intake ratio (Ca:Mg ratio), measured from the MgFFQ and the CaFFQ, was high in all participants, compared to an optimal ratio of 2:1 (Table 5) [20]. This was true across sexes, age groups, and BMI groups. Women had a higher Ca:Mg ratio, 3.47, than men, 2.82. Among age groups, the highest Ca:Mg ratio was in the 50 to 59 years age group, 3.64, and the lowest Ca:Mg ratio was in the 40 to 49 years age groups, 2.71. Among BMI groups, the highest Ca:Mg ratio was in individuals who were underweight, 3.69, and the lowest Ca:Mg ratio was in individuals who were obese, 2.65.

### 3.3. Agreement of MgFFQ and 14-Day Food Diary Magnesium Intake

The coefficient of variation (CV%) between the Mg intake measured by the MgFFQ and the 14-day food diary was similar across sexes, age groups, and BMI groups (Table 6). The highest CV% for all participants was 17.38%. Women and men had similar CV%, 17.38% and 17.17%, respectively. Among age groups, the highest CV% was in the 60 to 69 years age group, 22.84%, and the lowest CV% was in the 40 to 49 years age group, 11.40%. Among BMI groups, the highest CV% was in individuals who were underweight, 20.98%, and the lowest CV% was in individuals who were obese, 15.39%.

There were strong correlations between the Mg intake measured by the MgFFQ and the 14-day food diary across sexes, age groups, and BMI groups, indicating that the MgFFQ serves as a valid measure of daily Mg intake in adults (Table 7). The correlation between Mg intake from the FFQ and the 14-day food diary for all participants was *r* = 0.798 (*p* < 0.001). Men had a stronger correlation (*r* = 0.855, *p* < 0.001) between the two measures than women (*r* = 0.759, *p* < 0.001). Among age groups, the strongest correlations were in the 60 to 69 years age group (*r* = 1.000, *p* < 0.001) and the 40 to 49 years age group (*r* = 0.833, *p* < 0.001). Among BMI groups, the strongest correlation was in individuals who were overweight (*r* = 0.858, *p* < 0.001). In individuals who were underweight, there was a moderate correlation between the two measures, though it did not reach statistical significance (*r* = 0.714, *p* = 0.111).

## 4. Discussion

### 4.1. Summary of Results/Major Findings

Maintaining an adequate Mg status is important for overall health due to Mg’s role as a cofactor for over 300 enzymatic reactions [1,2,3]. The predominant way to improve Mg status is through dietary increases of Mg intake, though limited assessment tools exist to quickly and accurately measure dietary Mg intake to assess Mg adequacy [2,7]. Findings from our study overall showed that there was a strong positive agreement between the 33-item MgFFQ and the 14-day food diary in a sample of 135 adults of various sexes, ages, and BMI groups. This indicates that the MgFFQ is a valid method to quickly and accurately measure daily average Mg intake.

Strong agreements between the MgFFQ and the 14-day food diary were found in subsets of both women and men. The FFQ also appeared to be a valid tool for assessing daily average Mg intakes across all age groups, ranging from young adults ages 18 to 29 years, to older adults ages 60 to 69 years. The 40 to 49 years group, 50 to 59 years group, and 60 to 69 years group were small subsets of 8, 15, and 5 individuals, respectively, therefore requiring further study to generalize the validity of the MgFFQ in these specific populations.

Strong agreements between the MgFFQ and the 14-day food diary were also found across normal weight, overweight, and obese BMI groups. The agreement between the MgFFQ and the 14-day food diary in the underweight BMI group did not reach significance. However, this BMI group was a small subset of 6 individuals, therefore requiring further study to determine if a significant agreement exists in a larger sample of this specific population.

Overall, the strong positive agreement between the MgFFQ and the 14-day food diary in all participants, as well as in many of the subsets based on sex, age, and BMI groups support the use of the MgFFQ as a valid measurement tool that can be used to accurately assess average Mg intake in various demographic populations. Due to the low cost and quickness of administration of the MgFFQ, this tool can be used in both research and clinical settings, as an efficient and comprehensive representation of an individual’s Mg status. Compared to other methods of assessing Mg status, including cumbersome and expensive serum Mg measurements, and other FFQs such as the lengthy 139-item Nutrition Examination Survey (NHANES) Food Frequency Questionnaire (FFQ), the MgFFQ is a low-cost, quick to administer assessment tool that is specific to Mg intake [2].

### 4.2. Intake Ranges of Food Frequency Questionnaire Compared to Recommended Dietary Allowance

Median intake of Mg as measured by the MgFFQ for all participants (246.12 (178.27) mg/day) was approximately 40 mg/day lower than the intake of Mg measured by the 14-day food diary (280.80 (139.15) mg/day). This was true across both sexes. This difference in measured Mg intakes between the MgFFQ and the 14-day food diary may be due to the inherent structure of the MgFFQ, which contains 33 commonly consumed foods that are considered good sources of Mg in the diet. The MgFFQ does not capture minimal amounts of Mg that may be obtained from other food items, which are able to be captured by a 14-day food diary. This can potentially explain the small inconsistency of approximately 40 mg/day of measured Mg intake between these two measures.

Despite the small difference in measured average daily Mg intake from the MgFFQ compared to the 14-day food diary, average intakes of Mg as quantified by both measures were less than the RDA for sex and age on average for adult women and men in this study. The RDA for Mg for adults 31 to 70 years is 310 mg for women and 420 mg for men [2]. In our study, women consumed on average only 78% of their RDA for Mg, and men consumed on average only 59% of their RDA for Mg. This is consistent with previous reports that that in the United states, approximately 60% of adults do not reach their RDA for Mg intake [2,4].

Overall, poor Mg status in the United States is likely due to poor eating habits and low diet quality [2,21,22]. Magnesium is found in greatest amounts in foods that are minimally processed, such as nuts and legumes, green leafy vegetables, fruits, meat, and fish, which are often poorly consumed in the Westernized culture due to the high availability and consumption of ultra-processed, calorie-dense, nutrient-poor food choices [2,21]. Since poor Mg status has been associated with increased risk of different chronic diseases such as type 2 diabetes mellitus, a fast and efficient method to assess Mg intakes is prudent, in order to quickly identify and correct poor intake and reduce the risk of Mg deficiency [2].

### 4.3. Ca:Mg Ratio

In our study, the overall Ca:Mg ratio was 3.39 (2.11), with women having a higher Ca:Mg ratio compared to men. This may be related to the slightly higher percent of women, 18.7%, reporting Ca supplement use of higher amounts per day, compared to only 13.6% of men reporting supplemental Ca use per day. Among age groups, the 50 to 59 years group had the highest Ca:Mg ratio, and the 40 to 49 years group had the lowest Ca:Mg ratio. Both the 40 to 49 years group, and the 50 to 59 years group were small subsets of 4 and 6 individuals, respectively, and no individuals in the 60 to 69 years group and 70 to 75 years group had Ca:Mg ratio measurements, therefore requiring further study to generalize Ca:Mg ratios in these specific populations. Among BMI groups, the highest Ca:Mg ratio was found in individuals who were underweight, and the lowest Ca:Mg ratio was found in individuals who were overweight. The underweight group was a small subset of only 2 individuals, therefore requiring further study to generalize these findings.

In all sex, age, and BMI groups, the measured Ca:Mg ratio values were higher than the recommended Ca:Mg ratio of 2:1 [20]. It is important to note that the median intakes for Ca and Mg for all participants did not meet RDA, likely contributing to the poor Ca:Mg ratios found. This is reflective of the typical Western diet, which favors calorie-dense foods with low micronutrients content [21]. An imbalance of Ca:Mg ratio is associated with higher total mortality and increased risk of cardiovascular disease mortality based in previous studies [10,23]. Moreover, a high Ca:Mg ratio along with low Mg intake may exacerbate Mg deficiency. Previous evidence indicates that a high dietary calcium intake independently decreases intestinal absorption rates for both Ca and Mg, increases urinary excretion of Mg, and increases competition for the absorption of intestinal Mg due to a shared homeostatic regulating systems between Ca and Mg [10,24,25]. Poor Mg status has been previously linked to increased low-grade inflammatory stress, which is one of the risk factors for chronic diseases such as cardiovascular disease, type 2 diabetes mellitus, and certain types of cancer [2,23,26]. Therefore, there is a need to lower the dietary Ca:Mg ratio in the U.S. population.

### 4.4. Strength and Limitations

Nutrient-specific FFQs, such as the MgFFQ that was validated in this study, provide a quick and efficient means of measuring average intake of a nutrient such as Mg over an extended period of time. Compared to the 14-day food diary, a labor intensive method to collect Mg or other nutrient intake information, the MgFFQ is a quick method to capture typical dietary intake over several days or an extended period of time, therefore acting as a valuable tool for both clinicians and researchers to determine an individual’s typical dietary intake [19]. Additional strengths of this study include recruitment from a large sample of healthy adults with diverse sexes, ages, races/ethnicities, and body weight statuses.

Major limitations of this study include that the majority of participants were younger adults aged 18 to 39 years, due to most recruitment methods being conducted on a university campus. Additional research is needed to determine the validity of the MgFFQ in children under 18 years who were not examined in the present study, as well as in older adults ages 40 to 69 years, who were represented only with a limited number of participants in the present study. More so, while racial and sex variety did exist within the study sample, participants in these groups were uneven, potentially indicating better generalizability in females, and Caucasian and Asian participants, the largest gender and ethnic groups represented in this study sample. Finally, in all genders, ages, and ethnicities, additional research is needed to determine the correlation of the MgFFQ with weekday versus weekend day food consumption, as well as within specific food groups such as dairy and grains, since only a collective MgFFQ was assessed against a combined weekday and weekend 14-day food diary in this study.

Limitations of the MgFFQ inherently include its reliance on the memory of the participant for accuracy of information reported, as opposed to food diaries which are recorded in present time and therefore do not rely heavily on memory. While the labor intensity of the 14-day food diary may be suggested to be a limitation in this study due to the potential result of poor recording by the participants, this was accounted for by researchers reviewing all food diaries for accuracy and detail of recording prior to analysis. Specific food choices on the MgFFQ may also be considered a limitation in this study. The MgFFQ contained only major commonly consumed foods that are good sources of Mg, it did not fully represent all food sources that can provide Mg, including foods with trace amounts of Mg, as well as different cuisines of various cultures that provide Mg, that may contribute overall to measurement biases and limit the MgFFQ generalizability. Also contributing to this measurement bias, diet intake of alcohol consumption was not captured with the MgFFQ, though certain types of alcohol, such as beer, do contain a considerable amount of Mg [27]. Lastly, while the MgFFQ was considered a valid measurement to quantify average reported Mg intake per day in adults, no duplicate portion study or assessement of Mg intake and biochemical indicators of serum Mg statuses were examined or established in the present study, in order to determine if average Mg intake was associated with overall Mg status in the study sample.

## 5. Conclusions

Overall, the MgFFQ was considered a valid measure of daily Mg intake in adult men and women of varying ethnicities, ages, and weight statuses due to its strong agreement with Mg intake measured by the 14-day food diary. Future studies should expand the MgFFQ to include water, alcohol, and dietary supplements as potential sources of magnesium, as well as expand recruitment efforts to capture participants with a wider age range, gender distribution, and various ethnicities in order to further validate generalizability of the MgFFQ for use in these different populations. To assess Mg adequacy, future studies should also measure biochemical indicators of Mg status. Serum ionized or total Mg values can be assessed with Mg intake measured by the MgFFQ to determine overall sufficiency. Overall, the strong positive agreement between the MgFFQ and the 14-day food diaries validates that the MgFFQ serves as an accurate and quick method to estimate average daily Mg intake.

## Figures and Tables

**Table 1:** Demographic characteristics of study participants.

Variable	All Participants (*n* = 135)	Women (*n* = 91)	Men (*n* = 44)
Weight (kg)			
Mean ± SD	72.41 ± 17.54	66.37 ± 15.21	84.91 ± 15.43
Median (IQR)	70.45 (19.95)	65.91 (15.45)	85.00 (22.16)
Height (cm)			
Mean ± SD	169.53 ± 10.34	164.71 ± 7.41	179.49 ± 8.24
Median (IQR)	167.64 (13.97)	165.10 (12.70)	177.80 (12.70)
Age (years) ^a^			
Mean ± SD	31.25 ± 12.92	30.84 ± 12.93	32.70 ± 12.85
Median (IQR)	25 (12)	25 (11.5)	27 (17.5)
BMI (kg/m^2^)			
Mean ± SD	24.96 ± 4.78	24.37 ± 5.16	26.47 ± 3.87
Median (IQR)	24.37 (5.28)	23.89 (4.98)	26.1 (4.05)
Ethnicity (%)			
African American	7 (5.2)	4(4.4)	3 (6.81)
Asian	34 (25.2)	19 (20.9)	15 (34.09)
Caucasian	85 (63)	61 (67.0)	24 (54.54)
Hispanic	6 (4.4)	5 (5.5)	1 (2.27)
Other	3 (2.2)	2 (2.2)	1 (2.27)

Presented as means ± standard deviations for normally distributed continuous variables and medians (interquartile ranges) for not normally distributed continuous variables; frequency (percent) for categorical variables. SD, standard deviation. IQR, interquartile range. ^a^ In a subset of 134 individuals, due to one participant not providing age.

**Table 2:** Average macronutrient intake calculated from the 14-day food diary.

Variable	All Participants (*n* = 135)	Women (*n* = 91)	Men (*n* = 44)
Energy (kcal/day)	1592.18 ± 363.00	1530.26 ± 355.39	1733.06 ± 344.19
Protein (g/day)	77.42 ± 20.73	73.08 ± 19.81	87.29 ± 19.56
Carbohydrates (g/day)	182.76 ± 52.14	176.79 ± 50.22	196.00 ± 54.48
Total Fat (g/day)	60.90 (23.67)	58.47 (25.85)	68.25 (25.63)
Saturated Fat (g/day)	17.90 (8.85)	16.65 (8.68)	21.40 (8.70)
Fiber (g/day)	18.50 (11.38)	18.75 (10.63)	17.65 (14.28)
Sugar (g/day)	54.36 ± 21.98	53.89 ± 20.73	55.35 ± 24.59

Presented as means ± standard deviations for normally distributed variables and medians (interquartile ranges) in not normally distributed variables.

**Table 3:** Micronutrient intake from the 14-day food diary.

Variable	All Participants (*n* = 135)	Women (*n* = 91)	Men (*n* = 44)
Cholesterol (mg)	266.31 ± 118.30	244.85 ± 114.81	313.42 ± 113.34
Iron (mg)	13.30 (5.40)	12.24 (4.80)	14.25 (5.40)
Sodium (mg)	2466.68 (1064.50)	2322.50 (1069.96)	2718.50 (1184.50)
Phosphorus (mg)	1148.69 ± 292.59	1098.91 ± 262.40	1254.02 ± 326.85
Potassium (mg)	2243.24 ± 642.65	2193.19 ± 601.30	2346.84 ± 717.15
Vitamin A (RAE)	538.54 (327.35)	575.75 (327.54)	494.85 (320.25)
Vitamin C (mg)	65.50 (52.38)	70.20 (41.28)	55.53 (58.25)
Vitamin D (mcg)	1.24 (2.03)	1.31 (2.00)	1.12 (2.14)
Vitamin E (a-toc mg)	6.78 (5.58)	7.16 (5.96)	6.09 (4.98)
Vitamin K (mcg)	130.65 (148.89)	139.8 (145.88)	73.40 (134.38)
Thiamin (mg)	1.38 ± 0.41	1.31 ± 0.35	1.54 ± 0.48
Niacin (mg)	21.60 (7.77)	20.65 (7.18)	24.50 (11.06)
Riboflavin (mg)	1.76 ± 0.51	1.73 ± 0.50	1.82 ± 0.53
Folate (mcg)	363.18 (144.60)	351.15 (126.57)	366.80 (229.08)
Vitamin B6 (mg)	1.89 (0.89)	1.80 (0.87)	1.98 (0.93)
Vitamin B12 (mcg)	3.98 ± 1.69	3.79 ± 1.67	4.38 ± 1.69
Zinc (mg)	9.70 (3.61)	9.47 (3.11)	9.73 (5.16)

Presented as means ± standard deviations for normally distributed variables and medians (interquartile ranges) in not normally distributed variables. RAE, retinol activity equivalents; A-toc, alpha-tocopherol.

**Table 4:** Magnesium and calcium intakes calculated from the food frequency questionnaires and the 14-day food diary.

Variable	All Participants (*n* = 135)	Women (*n* = 91)	Men (*n* = 44)
Calcium FFQ (mg) ^a^	704.36 (486.55)	781.15 (534.66)	650.50 (533.75)
Calcium (mg) from 14d FD	627.71 (347.00)	612.10 (244.45)	642.07 (390.20)
Supplemental Calcium (mg) from FFQ	255.00 (300.00) ^b^	329.00 (400.00) ^c^	252.50 (267.50) ^d^
Magnesium FFQ (mg)	246.21 (178.27)	242.30 (126.11)	249.87 (233.74)
Magnesium (mg) from 14d FD	280.80 (139.15)	280.10 (116.90)	284.00 (206.00)

Magnesium and calcium intakes from the 14-day food diary and the food frequency questionnaires. Presented as means ± standard deviations for normally distributed variables and medians (interquartile ranges) in not normally distributed variables. 14d FD, 14-day food diary. ^a^ In a subset of 74 individuals. ^b^ In a subset of 17 individuals. ^c^ In a subset of 11 women. ^d^ In a subset of 6 men.

**Table 5:** Calcium to magnesium intake ratio from the food frequency questionnaires by sex, age, and body mass index.

Variable	Ca:Mg Ratio
All Participants (*n* = 69)	3.39 (2.11)
Sex	
Women (*n* = 40)	3.47 (1.93)
Men (*n* = 29)	2.82 (2.61)
Age ^a^	
18 to 29 years (*n* = 44)	3.40 (1.87)
30 to 39 years (*n* = 14)	2.87 (2.44)
40 to 49 years (*n* = 4)	2.71 (2.05)
50 to 59 years (*n* = 6)	3.64 (4.45)
60 to 69 years (*n* = 0)	-
70 to 75 years (*n* = 0)	-
Weight Status	
Underweight (*n* = 2)	3.69 (-)
Normal Weight (*n* = 36)	3.13 (2.52)
Overweight (*n* = 24)	3.42 (1.57)
Obese (*n* = 7)	2.65 (2.20)

Calcium to magnesium intake ratios (Ca:Mg Ratio) from the food frequency questionnaires. Presented as median (interquartile ranges) in not normally distributed variables. ^a^ In a subset of 68 individuals.

**Table 6:** Coefficient of variation between the magnesium food frequency questionnaire and 14-day food diary by sex, age, and body mass index.

Variable	Coefficient of Variation Percentage (CV%)
All Participants (*n* = 135)	17.38 (13.74)
Sex	
Women (*n* = 91)	17.38 (14.11)
Men (*n* = 44)	17.17 (12.70)
Age ^a^	
18 to 29 years (*n* = 84)	18.17 (14.08)
30 to 39 years (*n* = 21)	15.28 (14.69)
40 to 49 years (*n* = 8)	11.40 (10.54)
50 to 59 years (*n* = 15)	14.89 (18.92)
60 to 69 years (*n* = 5)	22.84 (10.02)
70 to 75 years (*n* = 1)	-
Weight Status	
Underweight (*n* = 6)	20.98 (14.97)
Normal Weight (*n* = 71)	17.82 (14.46)
Overweight (*n* = 42)	17.10 (14.11)
Obese (*n* = 16)	15.39 (13.23)

Magnesium intake from the FFQ and Mg intake from the 14-day food diary comparison of agreement. Presented as medians (interquartile ranges) in not normally distributed variables. ^a^ In a subset of 134 individuals.

**Table 7:** Correlations between magnesium food frequency questionnaire and 14-day food diary by sex, age, and body mass index.

Variable	*r*	*p*
All Participants (*n* = 133) ^a^	0.798	**<0.001**
Sex		
Women (*n* = 91) ^a^	0.759	**<0.001**
Men (*n* = 42) ^a^	0.855	**<0.001**
Age		
18 to 29 years (*n* = 84) ^a^	0.793	**<0.001**
30 to 39 years (*n* = 21) ^b^	0.805	**<0.001**
40 to 49 years (*n* = 8) ^b^	0.833	**<0.05**
50 to 59 years (*n* = 15) ^b^	0.697	**<0.05**
60 to 69 years (*n* = 5) ^b^	1.000	**<0.001**
70 to 75 years (*n* = 1)	-	-
Weight Status		
Underweight (*n* = 6) ^b^	0.714	0.111
Normal Weight (*n* = 71) ^a^	0.762	**<0.001**
Overweight (*n* = 42) ^a^	0.858	**<0.001**
Obese (*n* = 16) ^b^	0.675	**0.006**

Magnesium intake from the food frequency questionnaire and Mg intake from the 14-day food diary comparison of agreement via correlations. ^a^ Pearson’s correlations for sample sizes >25 and not normally distributed but meeting all other parametric assumptions; ^b^ Spearman’s correlation for sample sizes <25 and not normally distributed. Bolded values indicate significance of *p* < 0.05.

## Appendix A

This questionnaire determines your usual eating habits and foods high in magnesium.

Record how often you eat each food (daily, weekly, or monthly) and write the number in the corresponding column.

**Table A1:** Magnesium Food Frequency Questionnaire.

		How Often Do You Eat or Drink This Food or Beverage? (Fq)				
Food Source Containing Magnesium		Magnesium Content	# of Times/Day	# of Times/Week	# of Times/Month	Total Mg (mg/day)
Lentils, raw	1 cup	90				
Collards, cooked, boiled, drained, without salt	1 cup, chopped	40				
Kale, raw	1 cup	8				
Spinach, raw	1 cup	24				
Spinach, frozen, chopped or leaf, cooked, boiled, drained, without salt	½ cup	78				
Nuts, cashew nuts, dry roasted, with salt added	½ cup	178				
Pecans	½ cup, halves	60				
Seeds, pumpkin and squash seeds, whole, roasted, with salt added	1 cup	168				
Seeds, sunflower seed kernels, toasted, with salt added	1 cup	173				
Beans, black, mature seeds, canned, low sodium	1 cup	84				
Chickpeas (garbanzo beans, bengal gram), mature seeds, canned, solids and liquids	1 cup	65				
Salmon, wild, cooked, moist heat	3 oz.	30				
Mackerel, Atlantic, cooked, dry heat	3 oz.	82				
Fish, tuna, light, canned in water, drained solids	3 oz.	20				
Chicken, broiler or fryers, breast, skinless, boneless, meat only, cooked, grilled	3 oz.	29				
Peanut butter, smooth style, with salt	2 tbsp	54				
Peanut butter, chunk style, with salt	2 tbsp	51				
Banana, raw	1 small (6“ to 6–7/8” long)	27				
Strawberries, raw	1 cup, whole	19				
Blackberries, raw	1 cup	29				
Raisins, seedless	1 small box (1.5 oz)	14				
Quinoa, cooked	1 cup	118				
Bread, whole-wheat, commercially prepared	1 slice (32g)	24				
Bread, white, commercially prepared (includes soft bread crumbs)	1 slice (25g)	6				
Rice, brown, long-grain, cooked	1 cup	84				
Cereals, oats, regular and quick, not fortified, dry	1 cup	112				
Cereals, QUAKER, QUAKER MultiGrain Oatmeal, dry	1 cup	92				
Yogurt, fruit variety, nonfat	1 container (6 oz)	26				
Milk, whole, 3.25% milkfat, with added vitamin D	8 oz.	24				
Coffee, brewed from grounds, prepared with tap water	8 oz.	7				
Orange juice drink	8 oz.	7				
Candies, Special Dark Chocolate Bar	1 bar (73g)	23				
Avocado, raw, all commercial varieties	1 cup, cubes	44				

Magnesium intake from the food frequency questionnaire.

## References

[1] Elin R.J. (1994). Magnesium: The fifth but forgotten electrolyte. Am. J. Clin. Pathol..

[2] Institute of Medicine (US) Standing Committee on the Scientific Evaluation of Dietary Reference Intakes (1997). The National Academies Collection: Reports funded by National Institutes of Health. Dietary Reference Intakes for Calcium, Phosphorus, Magnesium, Vitamin D, and Fluoride.

[3] Takaya J., Higashino H., Kobayashi Y. (2004). Intracellular magnesium and insulin resistance. Magnes. Res..

[4] Nielsen F.H. (2010). Magnesium, inflammation, and obesity in chronic disease. Nutr. Rev..

[5] Rude R.K., Wei L., Norton H.J., Lu S.S., Dempster D.W., Gruber H.E. (2009). TNFalpha receptor knockout in mice reduces adverse effects of magnesium deficiency on bone. Growth Factors.

[6] Deng X., Song Y., Manson J.E., Signorello L.B., Zhang S.M., Shrubsole M.J., Ness R.M., Seidner D.L., Dai Q. (2013). Magnesium, vitamin D status and mortality: Results from US National Health and Nutrition Examination Survey (NHANES) 2001 to 2006 and NHANES III. BMC Med..

[7] Jahnen-Dechent W., Ketteler M. (2012). Magnesium basics. Clin. Kidney J..

[8] Gullestad L., Midtvedt K., Dolva L.O., Norseth J., Kjekshus J. (1994). The magnesium loading test: Reference values in healthy subjects. Scand. J. Clin. Lab. Investig..

[9] Razzaque M.S. (2018). Magnesium: Are We Consuming Enough?. Nutrients.

[10] Kelsay J.L. (1987). Effects of fiber, phytic acid, and oxalic acid in the diet on mineral bioavailability. Am. J. Gastroenterol..

[11] Torre M., Rodriguez A.R., Saura-Calixto F. (1991). Effects of dietary fiber and phytic acid on mineral availability. Crit. Rev. Food Sci. Nutr..

[12] Behar J. (1975). Effect of calcium on magnesium absorption. Am. J. Physiol..

[13] Willett W. (1998). Nutritional Epidemiology.

[14] Willett W.C., Sampson L., Stampfer M.J., Rosner B., Bain C., Witschi J., Hennekens C.H., Speizer F.E. (1985). Reproducibility and validity of a semiquantitative food frequency questionnaire. Am. J. Epidemiol..

[15] National Health ann Nutrition Examination Survey: Analytic Guidelines, 2011–2014 and 2015–2016. https://wwwn.cdc.gov/nchs/data/nhanes/2011-2012/analyticguidelines/analytic_guidelines_11_16.pdf.

[16] NHANES Food Questinnaire. https://www.cdc.gov/nchs/data/nhanes/nhanes_03_04/tq_fpq_c.pdf.

[17] International Osteoporosis Foundation Calcium Calculator. https://www.iofbonehealth.org/calcium-calculator.

[18] Cade J., Thompson R., Burley V., Warm D. (2002). Development, validation and utilisation of food-frequency questionnaires—A review. Public Health Nutr..

[19] Centers for Disease Control and Prevention Assessing Your Weight. https://www.cdc.gov/healthyweight/assessing/index.html.

[20] Rosanoff A. (2010). Rising Ca: Mg intake ratio from food in USA Adults: A concern?. Magnes. Res..

[21] Martinez Steele E., Baraldi L.G., Louzada M.L., Moubarac J.C., Mozaffarian D., Monteiro C.A. (2016). Ultra-processed foods and added sugars in the US diet: Evidence from a nationally representative cross-sectional study. BMJ Open.

[22] Agarwal S., Reider C., Brooks J.R., Fulgoni V.L. (2015). Comparison of prevalence of inadequate nutrient intake based on body weight status of adults in the United States: An analysis of NHANES 2001–2008. J. Am. Coll. Nutr..

[23] Rosanoff A., Weaver C.M., Rude R.K. (2012). Suboptimal magnesium status in the United States: Are the health consequences underestimated?. Nutr. Rev..

[24] Dai Q., Shu X.O., Deng X., Xiang Y.B., Li H., Yang G., Shrubsole M.J., Ji B., Cai H., Chow W.H. (2013). Modifying effect of calcium/magnesium intake ratio and mortality: A population-based cohort study. BMJ Open.

[25] Dai Q., Zhu X., Manson J.E., Song Y., Li X., Franke A.A., Costello R.B., Rosanoff A., Nian H., Fan L. (2018). Magnesium status and supplementation influence vitamin D status and metabolism: Results from a randomized trial. Am. J. Clin. Nutr..

[26] Nielsen F.H. (2018). Magnesium deficiency and increased inflammation: Current perspectives. J. Inflamm. Res..

[27] Gorinstein S., Zemser M., Libman I., Trakhtenberg S., Caspi A. (1998). Effect of beer consumption on plasma magnesium: Randomized comparison with mineral water. J. R. Soc. Med..

